# Climate-driven tipping-points could lead to sudden, high-intensity parasite outbreaks

**DOI:** 10.1098/rsos.140296

**Published:** 2015-05-20

**Authors:** Naomi J. Fox, Glenn Marion, Ross S. Davidson, Piran C. L. White, Michael R. Hutchings

**Affiliations:** 1 Disease Systems Team, SRUC, King's Building, West Mains Road, Edinburgh EH9 3JG, UK; 2 Biomathematics and Statistics Scotland, King's Buildings, West Mains Road, Edinburgh EH9 3JZ, UK; 3 Department of Environment, University of York, Heslington, York YO10 5DD, UK

**Keywords:** climate change, helminth, livestock, nematode, parasite, temperature

## Abstract

Parasitic nematodes represent one of the most pervasive and significant challenges to grazing livestock, and their intensity and distribution are strongly influenced by climate. Parasite levels and species composition have already shifted under climate change, with nematode parasite intensity frequently low in newly colonized areas, but sudden large-scale outbreaks are becoming increasingly common. These outbreaks compromise both food security and animal welfare, yet there is a paucity of predictions on how climate change will influence livestock parasites. This study aims to assess how climate change can affect parasite risk. Using a process-based approach, we determine how changes in temperature-sensitive elements of outbreaks influence parasite dynamics, to explore the potential for climate change to influence livestock helminth infections. We show that changes in temperate-sensitive parameters can result in nonlinear responses in outbreak dynamics, leading to distinct ‘tipping-points’ in nematode parasite burdens. Through applying two mechanistic models, of varying complexity, our approach demonstrates that these nonlinear responses are robust to the inclusion of a number of realistic processes that are present in livestock systems. Our study demonstrates that small changes in climatic conditions around critical thresholds may result in dramatic changes in parasite burdens.

## 1. Introduction

The distribution and abundance of livestock helminths (parasitic worms) has been shifting and increasing in temperate regions [1–3], with climate change implicated as one of the main drivers [1,4,5]. With potential for further climate-driven increases in parasite prevalence and intensity, and the consequent welfare and economic implications, there is a need to understand the impacts of climate change on macro-parasite transmission. Helminth infections in temperate regions were historically limited to species better adapted to colder climes e.g. *Ostertagia ostertagi*, *Teladorsagia circumcincta*, *Cooperia* spp., *Trichostrongylus* spp. and *Nematodirus* spp. However, helminth abundance and species composition have changed in temperate regions [1,2,6], with an increase in tropically adapted species such as *Haemonchus contortus*, which typically dominates in regions with hot summers [7].

Although the range of *H. contortus* has expanded, outbreak intensity remains low across much of its new range [6,8]. With parasite burdens not high enough to cause clinical cases in most of this new range, few stakeholders are taking proactive measures to limit parasite spread, remaining unconcerned about gradual range expansions. However, heavy infections are now occurring sporadically in newly affected areas and pathological heamonchosis cases are becoming an increasing problem for farmers [6]. Changing outbreak patterns are thus physically and economically damaging, as parasitism is characterized by weight loss, lower milk yield, condition loss, abortion and infertility, with heavy infections causing host mortality [9,10]. It is not yet understood what is driving this pattern of increasingly widespread low level infection with sporadic high-intensity outbreaks, although changing climate is a possible driver.

Climate change will impact various elements of helminth growth and transmission, and the influence of abiotic conditions on key life cycle parameters has been extensively studied [7,11]. Temperature is the predominant influence on free-living stages; increased temperatures drive an increase in parasite development rate for a majority of livestock helminths [12,13]. However, extreme temperatures can be inimical to larval survival [13], with thermal tolerance ranges varying between parasite species [7]. In addition to influencing larval survival and development within a grazing season, temperature also affects over-winter survival and thus availability of infective larvae at the start of the following grazing season. It is these larvae that initiate infections early in the year when naive hosts are turned out to pasture [14].

A number of studies have aimed to link past changes in helminth distributions and abundance with climate change [1–3,15], yet lack of long-term active surveillance data means the relationship between climate and outbreak patterns has yet to be quantified [16]. Given the lack of data, we address this issue using a process-based modelling approach to explore the potential for climate change to influence the dynamics of livestock helminth infections. The basis of this approach is that where key processes are sufficiently well understood, models can be used to explore the potential behaviour of a system under new conditions. Such an approach allows predictions of likely outcomes under future scenarios, and assessment of the robustness of such conclusions under a range of assumptions.

Our approach is based on a generic model of helminth transmission dynamics developed by Roberts & Grenfell [17] which has previously been shown to recreate general helminth infection patterns observed in managed grazing systems [17–19]. One criticism of this work is that the exposure sub-model fails to account for spatial and temporal heterogeneity in parasite risk generated by the interaction of variations in host burden, individual grazing and avoidance behaviour and the dynamics and spatial distribution of parasites. It is important to incorporate these elements when exploring the effects of changing larval development times, as the risk to hosts depends jointly on the rate at which parasite larvae become infective and the rate at which a patch is grazed. We therefore combine this helminth transmission dynamics model with a model describing the spatial and temporal dynamics of host grazing. This grazing model is based on empirical rules of thumb describing grazing and faecal avoidance behaviour [20–24] and has been shown to reproduce emergent patterns observed at the field scale in experimental systems [22,25–28].

Using this process-based approach, we aim to determine how changes in temperature-sensitive elements of outbreaks influence macro-parasite dynamics, to explore the potential for climate change to influence livestock helminth infections. Specifically, we explore the influence of: (i) changing development rates of parasites’ free-living stages; (ii) changing death rates of free-living stages; (iii) host grazing behaviours under a changing climate; and (iv) over-winter survival of the parasites’ free-living stages.

## 2. Material and methods

The application of a mechanistic model that incorporates key elements of parasite outbreaks allows us to explore how changes in climate-sensitive parameters influence parasite intensity. A non-spatial, population-level model (based on that developed by Roberts & Grenfell [17]) is initially used to explore the fundamental influence of changing key temperature-sensitive parameters (larval development and survival) on parasite burdens. Through an extension of this model, these fundamental patterns are then explored within the context of a wider system of interacting processes that have been shown to influence outbreak dynamics [29]. These models represent nematode parasites transmitted via the faecal oral route, within a managed livestock production system. Both models were simulated as stochastic, discrete state-space event-based Markov processes using the Gillespie algorithm [30].

### 2.1. Non-spatial, population-level model

The non-spatial, population-level model encapsulates the dynamics of directly transmitted gastro-intestinal nematode infection in managed ruminant populations. It is based on that proposed by Roberts & Grenfell [17] and later developed by Marion *et al.* [19], who used a stochastic formulation to better reflect the variability in biological systems. Roberts & Grenfell [17] distilled helminth transmission down to three variables (adult parasites in the host, parasite larvae on pasture and host immunity), and were able to recreate the general infection patterns observed in managed grazing systems. However, the inclusion of additional elements was required to address how changing temperatures could affect outbreak dynamics. Helminth parasites can spend a large part of their life cycle outside of the definitive host, and survival and development of the free-living stages is affected by changes in temperature. Hence, exploring effects of temperature changes on outbreak patterns requires inclusion of survival and development of the parasites’ free-living stages.

In our model, the principal features of parasite transmission are represented by four state variables: mean population of free-living pre-infective larvae, *l*, mean population of free-living infective larvae, *L*, mean intensity of adult parasites in the host, *A* and the level of acquired immunity, *r*. The model incorporates the probability that any egg will hatch, *q*, the rate at which host immunity is lost in the absence of infection, *σ*, the probability of larvae dying, *ρ*, contact rate, *β* and the rate of larval development on pasture, *α*, as well as the rate of egg production, *λ*(*r*), adult mortality rate, *μ*(*r*) and the probability ingested larvae become adults, *p*(*r*), which are functions of the level of immunity in the host. Tables 1–3 summarize all states, parameters and events in the non-spatial, individual-level model.

**Table 1.** Summary of states in the non-spatial, population-level model.

states	notation
free-living pre-infective larvae	*l*
free-living infective larvae	*L*
adult parasites in host	*A*
acquired immunity	*r*

**Table 2.** Summary of parameters in the non-spatial, population-level model. (All parameters are in units of min^−1^, except *p*, *q* and *r* which are dimension free.)

parameter	notation	value
death of larvae	*ρ*	0.000015 [11,31]
contact rate	*β*	6.9×10^−7^[17]
larval development	*α*	0.00005 [11,13,32,33]
egg hatch probability	*q*	0.35 [17]
loss of immunity	*σ*	1.9×10^−8^[17]
rate of egg production	*λ*(*r*)	2 [17]
probability ingested larvae become adults	*p*(*r*)	0.65 [17]
death of adult larvae	*μ*(*r*)	0.0000047 [17]

**Table 3.** Summary of events for non-spatial, population-level model.

event	rate	change in state space
uptake	*βL*	*L*→*L*−1
		*r*→*r*+1
		*A*→*A*+1, with probability *p*(*r*)
adult death	*μ*(*r*)*A*	*A*→*A*−1
fecundity	*q* *λ*(*r*)*A*	*l*→*l*+1
immunity loss	*σr*	*r*→*r*−1
l death	*ρl*	*l*→*l*−1
L death	*ρL*	*L*→*L*−1
larval development	*αl*	*l*→*l*−1
		*L*→*L*+1

The influence of changes in temperature-sensitive elements on outbreak dynamics (larvae survival and development) were first explored with this simple, non-spatial, population-level model. For gastro-intestinal nematodes of herbivores, development times vary from less than one week to over a month [11,13,32,33]. The development rate of parasites on pasture, from non-infective to infective stages, increases with temperature [12,34,35]. Mean temperatures are projected to increase under climate change, and an increase in extreme weather events is also expected [36]. This increase in temperature will increase the development rates of the parasites’ free-living stages. Here, we explore the potential impacts of increasing temperatures on parasite burden implicitly through changes in the development rate of the parasite's free-living stages. Larval development rates were varied to give on-pasture development times ranging from no development to a fast development time of around 3 days (development rate of 0.0002 min^−1^; figure 2). Larval death rates were varied to give average on-pasture survival times ranging from around 3 days (death rate of 0.0002 min^−1^) to around 35 days (death rate of 0.00002 min^−1^; figure 3). Each scenario was repeated over 10 realizations to account for the stochastic nature of the model.

**Figure 1.**
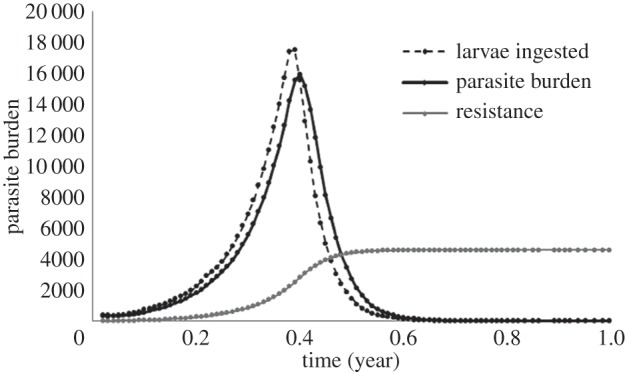
Parasite dynamics over one grazing season. Host–parasite burden, L3 ingested per day and host resistance level over one grazing season, for one run of the non-spatial, individual-based model, using the standard parameter values detailed above.

When presenting the results, peak parasite burden is used as a measure of infection, as host morbidity and mortality are directly proportional to parasite intensity [37]. However, a host can be affected by both parasite intensity and duration of infection. To determine the usefulness of this measure as a reliable indicator of disease levels, both the peak parasite intensity and the cumulative exposure over the grazing season, measured by integrating the infection curve shown in figure 1, were calculated for the scenarios detailed above. Over the range of simulations, both measures provided qualitatively similar results. Peak parasite intensity is used as a measure of infection here as it is a more intuitive measure than the area under the curve, and can be compared to empirical data. If cumulative burden was chosen instead as a measure of parasitism, the trends shown in the results, and the conclusions, would remain the same.

### 2.2. Spatially explicit, individual-level model

The non-spatial, population-level model detailed above captures the basic processes in helminth infections and highlights fundamental trends in outbreak dynamics that result from changes in larval survival and development. However, it is imperative to consider climate-sensitive elements of transmission within the context of a wider system of interacting processes.

Although governed by basic rules, transmission is a complex process, and it has been shown that host grazing behaviour and spatial effects are important in outbreak dynamics [29]. While larval survival and development determine the temporal pattern of availability of infective larvae on pasture, ultimately host grazing behaviour determines when these free-living stages are ingested. Consequently, the framework is extended to explore the robustness of the results to the inclusion of grazing behaviours and spatial effects.

The complex grazing patterns seen in livestock systems (and the times at which hosts encounter free-living parasites) have been shown to be driven by a number of simple grazing rules. At the individual bite scale, hosts seek out patches of tall sward [38,39] whilst avoiding taking bites of faecally contaminated sward [21]. At the field scale, the host's preference for grazing tall swards and avoiding faecally contaminated swards creates a heterogeneous gap and tussock mosaic. Consequently, grazing livestock are faced with a nutrition versus parasitism trade-off, as ungrazed contaminated sward grows taller and uncontaminated sward is depleted. The model developed by Marion *et al*. [22,25–28] used these grazing rules to recreate grazing behaviours observed at multiple scales. At the bite scale host grazing decisions regarding faecal avoidance and sward selection matched empirical observations of grazing behaviour, while at the field scale resource distribution matched that observed in set stocked grazing systems [20]. This element of the model is described in detail in [22,25–28].

In Fox *et al.* [29], this model was further developed to incorporate helminth parasite transmission. This work demonstrated that host grazing behaviour affects both the timing and magnitude of peak parasite burdens [29]. Fox *et al.* [29] also demonstrated that the spatial clumping of parasites on pasture shown in the model matches empirically measured distributions of parasites [40]. If this spatial heterogeneity in risk is not incorporated, then outbreak severity can be underestimated [29]. The ability if the model to recreate these grazing behaviours and the spatio-temporal variation in risk and resources is fundamental, as it determines the timing at which hosts contact the free-living parasite larvae.

To determine the impact of host grazing behaviour when exploring changes in survival and development of the parasites’ free-living stages we use the spatially explicit, individual-based model developed by Fox *et al.* [29]. This model is a further development of the non-spatial model but also incorporates the wider elements of the transmission process. These include development of parasites within hosts, spatial heterogeneity of resources, pathogens and perceived pathogen risk, and host grazing behaviours, in addition to all elements incorporated in the simpler model described above.

The spatially explicit, individual-based model incorporates the key elements of pathogen population dynamics on pasture and in the host in addition to host grazing behaviour. The model was developed by Marion *et al*. [25] and Fox *et al*. [29]. A cohort of *D* animals (labelled *k*=1…*D*) move around a lattice of *N* patches (labelled *i*=1…*N*), making grazing decisions based on the sward height *h*_*i*_ at that patch and the level of faecal contamination *f*_*i*_. The patch and animal state variables are outlined in table 1. All state variables within the model are assumed to be integers.

The rate of movement from patch *i* to patch *j* is modelled as (*v*/*z*(*i*))*F*(*i*,*j*)*h*_*j*_, where *v* is the intrinsic movement rate and *h*_*j*_ is the sward height at patch *j*, using the normalization factor

$$z(i)=\sum_{j\in N_{i}}F(i,j).$$

The search kernel *F*(*i*,*j*) follows the power-law *F*(*i*,*j*)=|*i*−*j*|^−*α*^ in which |*i*−*j*| is the Euclidean distance between patch *i* and *j*. Sward growth is modelled logistically with the rate of increase at patch *i* given by

$$\gamma h_{i}\left(1-\frac{h_{i}}{h_{\max}}\right),$$

where *γ* is the intrinsic growth rate of the sward, and $h_{\max}$ is the maximum sward height attainable. A self-limiting growth function is used to prevent exponential growth of ungrazed patches. This logistic growth function also allows for the variation in grass growth rate with changing sward height [41]. Sward height is measured in units of bite size, where one unit is equal to one cattle bite of 0.001 m^2^ [42], and each patch is set at 0.5 m^2^ as this is the typical area affected by cattle faecal contamination and the refusal area around it [42]. Complete removal of grass from individual patches is prevented, as grazing livestock typically graze sward to a minimal level. For example, cattle typically graze sward down to 2 cm [42], leaving a portion of ungrazed sward to recover. This is reflected here, with an ungrazeable portion of grass (*h*_0_) being considered when calculating the probability of a bite occurring at a certain patch. Each 0.5 m^2^ patch contains a minimum of 50 bites of forage, each patch has an initial sward height of 200 bites and a maximum sward height of 400 bites. The sward growth rate is calculated to provide a set stock scenario where sward growth is equal to an overall herbivore intake of 30 000 bites per day [42]. The bite rate function leads to a linear relationship between number of bites per visit to a patch, and the sward height at that patch upon arrival. This is consistent with the behavioural observation that bite depth is proportional to sward height [43].

The sward height of a given patch is reduced by *B* when an animal grazes at that location, while the stomach content *s*_*k*_ of the corresponding animal is increased by one unit of size *B*. An individual takes a bite on its current patch at a rate

$$\beta (h_{i}-h_{0}){\text{e}}^{-\mu f_{i}(a_{k}+A_{k})},$$

where *f*_*i*_ represents the level of faecal contamination at patch *i*, *μ* is the level of faecal avoidance, *a*_*k*_+*A*_*k*_ is the total number of parasites in host *k*, and *h*_*o*_ is the minimum grazable portion in each patch. Thus, the bite rate is monotonically decreasing with the amount of faecal contamination and level of avoidance. The model also includes a daily intake requirement *R*_*k*_ for each animal [44]. The intake of each animal accumulates until its requirement *R*_*k*_ is reached and is reset at the end of each day.

Each patch (labelled *i*=1…*N*) is assigned a number *l*_*i*_ of pre-infective larvae as well as a number *L*_*i*_ of infective L3 stage larvae. Similarly, within each host (labelled *k*=1…*D*) separate variables *a*_*k*_, *A*_*k*_ and *e*_*k*_ are introduced for the number of immature parasites, mature parasites and eggs, respectively.

When an animal takes a bite of size *B*, the number of non-infective (*l*_*i*_) and infective larvae (*L*_*i*_) on its current patch, decrease by

$$\frac{B}{h_{i}}l_{i}\;\text{and}\;\frac{B}{h_{i}}L_{i}.$$

When an animal takes a bite of size *B*, the number of immature parasites in host *k*,*a*_*k*_, increases by

$$\theta (r_{k})\frac{B}{h_{i}}L_{i},$$

where *θ* is the probability of ingested L3 larvae establishing and becoming immature larvae in the host, and is a monotonic non-increasing function of *r*, representing the detrimental effect of resistance on parasite establishment. When infective larvae are ingested, the resistance *r*_*k*_ of host *k* increases by

$$\frac{L_{i}}{h_{i}}B\psi ,$$

where *ψ* is a resistance gain coefficient. *r*_*k*_ also increases as a function of the current parasite burden, at rate (*a*_*k*_+*A*_*k*_) *η*, where *η* is a second resistance gain coefficient. Death of immature parasites in the host occurs at a rate *ζa*_*k*_. Immature parasites develop into mature, egg producing adult parasites at a rate *χa*_*k*_. Death of adults in host *k* occurs at rate *τ*(*r*_*k*_)*A*_*k*_, where *τ*(*r*_*k*_)>0 is a monotonic non-decreasing function which models the influence of acquired immunity on parasite mortality in the host. In the absence of exposure resistance in host *k* decays at rate *σr*_*k*_, following the model design of Roberts & Grenfell [17] and Marion *et al.* [19].

The number of eggs, *e*_*k*_, in host *k* is affected by egg production from the dioecious parasites within the host at a rate

$$\frac{\lambda (r_{k})A_{k}}{2},$$

where *λ* (*r*_*k*_), the rate of egg production per adult parasite, is a monotonic non-increasing function of *r*_*k*_.

The rate of defecation for an individual in its current patch is *f*dep(*s*_*k*_−*s*_0_)*Θ*(*s*_*k*_−*s*_0_) where the Heaviside function *Θ*(*s*_*k*_−*s*_0_) is unity if the stomach contents, *s*_*k*_, are greater than the faecal deposit size, *s*_0_, and is otherwise zero. Each faecal deposit is equivalent to 2000 bites of sward to reflect the cattle defecation rate of approximately 15 times per day [42]. When a defecation event occurs, *e*_*k*_ decreases by *s*_0_/*s*_*k*_×*e*_*k*_ and the number of pre-infective larvae in patch *i*,*l*_*i*_, increases by the same quantity. The non-infective larvae develop into infective larvae on pasture at a rate of *ɛl*_*i*_. The decay rate for faecal contamination at patch *i* is *φf*_*i*_, and is parametrized so that complete degradation occurs three months after deposition [45], and the death rates of L and L3 larvae are *ωl*_*i*_ and *ρL*_*i*_, respectively. The stochastic model is simulated on the state-space variables (table 4) using the events and associated rates described in table 5, following the Gillespie algorithm [46]. Model parameters are listed in table 6.

**Table 4.** Summary of state variables in the spatial, individual-based model.

patch states	notation
coordinates of patch *i*	(*x*_*i*_, *y*_*i*_)
sward height at patch *i*	*h*_*i*_
faecal contamination at patch *i*	*f*_*i*_
pre-infective larvae at patch *i*	*l*_*i*_
infective L3 larvae at patch *i*	*L*_*i*_

animal states	notation
location of animal *k*	*i*_*k*_
immune response of animal *k*	*r*_*k*_
immature parasites in animal *k*	*a*_*k*_
mature parasites in animal *k*	*A*_*k*_
parasite eggs in animal *k*	*e*_*k*_
stomach contents of animal *k*	*s*_*k*_
faecal deposit size	*s*_0_

**Table 5.** Summary of events in the spatial, individual-based model, for patch *i* and host *k*.

each patch event	rate r_*ei*_	change in state variables
growth of sward at patch *i*	$\gamma h_{i}(1-(h_{i}/h_{\max}))$	*h*_*i*_→*h*_*i*_+1
development of larvae at patch *i*	*ɛl*_*i*_	*l*_*i*_→*l*_*i*_−1
		*L*_*i*_→*L*_*i*_+1
death of pre-infective larvae at patch *i*	*ωl*_*i*_	*l*_*i*_→*l*_*i*_−1
death of infective L3 at patch *i*	*ρL*_*i*_	*L*_*i*_→*L*_*i*_−1
decay of faeces at patch *i*	*φf*_*i*_	*f*_*i*_→*f*_*i*_−1

animal event	rate r_*ek*_	change in state variables
bite at current patch *i*, potential	*β*(*h*_*i*_−*h*_0_)e^−*μ*_*k*_*f*_*i*_(*a*_*k*_+*A*_*k*_)^	*h*_*i*_→*h*_*i*_−1
ingestion of infective and pre-infective larvae,		*L*_*i*_→*L*_*i*_−(*B*/*h*_*i*_)*L*_*i*_
potential establishment of infective larvae		*l*_*i*_→*l*_*i*_−(*B*/*h*_*i*_)*l*_*i*_
and gain in immunity		*s*_*k*_→*s*_*k*_+1
		*r*_*k*_→*r*_*k*_+(*B*/*h*_*i*_)*l*_*i*_
		*a*_*k*_→*a*_*k*_+*θ*(*r*_*k*_)(*B*/*h*_*i*_)*L*_*i*_
death of immature adults in host *k*	*ζa*_*k*_	*a*_*k*_→*a*_*k*_−1
maturity of adults in host *k*	*χa*_*k*_	*a*_*k*_→*a*_*k*_−1
		*A*_*k*_→*A*_*k*_+1
death of adults in host *k*	*τ*(*r*_*k*_)*A*_*k*_	*A*_*k*_→*A*_*k*_−1
gain of immunity in host *k* due to parasite burden	(*a*_*k*_+*A*_*k*_)*η*	*r*_*k*_→*r*_*k*_+1
loss of immunity in host *k*	*σr*_*k*_	*r*_*k*_→*r*_*k*_−1
egg production in host *k*	*λ*(*r*_*k*_)*A*_*k*_/2	*e*_*k*_→*e*_*k*_+1
defecation by host *k*	*f* dep (*s*_*k*_−*s*_0_)*Θ*(*s*_*k*_−*s*_0_)	*e*_*k*_→*e*_*k*_−((*s*_0_/*s*_*k*_)*e*_*k*_)
		*e*_*k*_→*e*_*k*_+((*s*_0_/*s*_*k*_)*e*_*k*_)
		*s*_*k*_→*s*_*k*_−*s*_*o*_
		*f*_*i*_→*f*_*i*_+*s*_*o*_
movement of animal *k*	*v*/(*z*(*i*))*F*(*i*,*j*)*h*_*j*_	*i*_*k*_=*i*→*i*_*k*_=*j*

**Table 6.** Summary of parameters in the spatial, individual-based model. (All parameters are in units of min^−1^, except *p*, *q* and *r* which are dimension free.)

parameter	notation	value
patch		
intrinsic growth rate of sward	*γ*	0.00004 [44]
development rate of L to L3 larvae	*ɛ*	0.00005 [11,13,32,33]
death rate of pre-infective larvae (L)	*ω*	0.0001 [11,32]
death rate of L3 larvae	*ρ*	0.000015 [11,31]
decay of faeces	*φ*	0.00001776 [45]
animal		
bite rate	*β*	0.01 [42]
faecal avoidance coefficient	*μ*	5 [47,48]
death of immature larvae in host	*ζ*	0.00005 [29]
maturity of larvae in host	*χ*	0.00003 [11]
rate of resistance loss	*σ*	1.9×10^−8^[17]
resistance gain coefficient 1	*ψ*	0.25 [29]
resistance gain coefficient 2	*η*	0.025 [29]
death rate of adult larvae in host	*τ*	0.00002 [11]
rate of egg production of adult parasite	*λ*(*r*_*k*_)	2 [11]
intrinsic movement rate	*v*	0.015 [49]
probability of ingested L3 larvae establishing as adults	*θ*(*r*_*k*_)	0.4 [11]

For non-climate-driven parameters, parameter values were taken from Fox *et al.* [18]. When considering the importance of host grazing behaviour (figure 4), larval development times varied from 10 weeks to one week. Faecal avoidance for each animal was initially set to no avoidance (figure 4) and then to realistic levels of avoidance (figures 4 and 5). With realistic levels of faecal avoidance, the model reproduces the livestock grazing behaviour that is empirically observed at multiple scales [23,24,27,50]. The starting condition of the simulation was representative of naive hosts being released onto contaminated pasture. Each simulation was initialized with uninfected hosts on a pasture with 24 000 infective larvae, distributed over 0.3% of randomly selected patches to reflect the aggregated distribution of larvae on pasture [40]. Each scenario was repeated over 10 realizations to account for the stochastic nature of the model.

When considering the importance of over-wintering larvae (figure 5), larvae development and death times varied from 10 weeks to one week. Simulations were initialized with uninfected hosts on pasture with low (12 000), medium (24 000) and high (48 000) numbers of infective larvae, distributed over 0.3% of randomly selected patches on the field.

## 3. Results

### 3.1. Outbreak dynamics

Using values outlined in table 2, the model successfully reproduces parasite dynamics observed in grazing livestock systems [17,51–54], with one run of the model shown in figure 1. The introduction of naive hosts onto contaminated pasture leads to a rapid rise in ingestion and establishment of infective parasite larvae, leading to a rise in parasite burden. The consequent increase in immunity then deleteriously affects parasite establishment and fecundity, leading to the subsequent decline in parasite burden. Figure 1 illustrates the distinctive peak which is referred to here as the peak parasite burden.

### 3.2. Larval development

The development rate of parasites on pasture, from pre-infective to infective stages, is well known to rise with increasing temperatures [12,34,35]. For gastro-intestinal nematodes of herbivores, development times vary from less than one week to over five months [11,13,32,33]. To investigate how temperature-driven changes in development rate influence host–parasite burdens, simulations were run using the non-spatial, population-level model, across a range of values of the development rate parameter.

**Figure 2.**
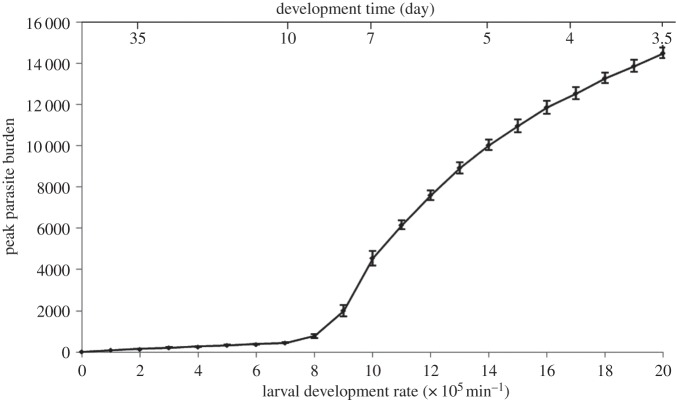
Peak parasite burden over different larval development rates (±s.d.).

Simulations show that increasing development rates results in a nonlinear increase in parasite burdens, with a distinct tipping-point (figure 2).

### 3.3. Larval death rate

**Figure 3.**
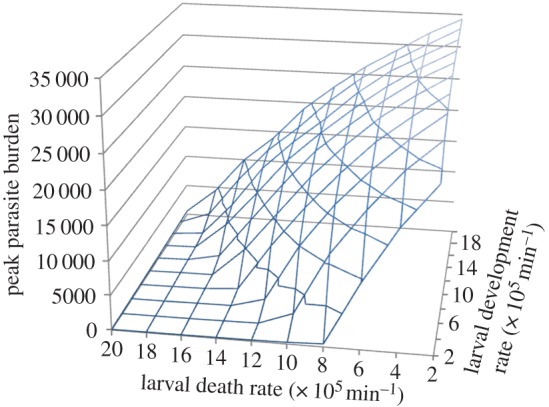
Influence of larvae death and development rates on peak parasite burden.

Increased summer temperatures can affect the death rates of free-living larvae [7]. Three-dimensional plots exploring changes in both larval development and death rates (again using the non-spatial, population-level model) show how these parameters influence parasite levels (figure 3).

Figure 3 illustrates that parasite burden is dependent on the relationship between parasite development and death rates, with the tipping-point for a specific development rate being pushed back as death rates increase.

### 3.4. The importance of livestock grazing behaviour and management

**Figure 4.**
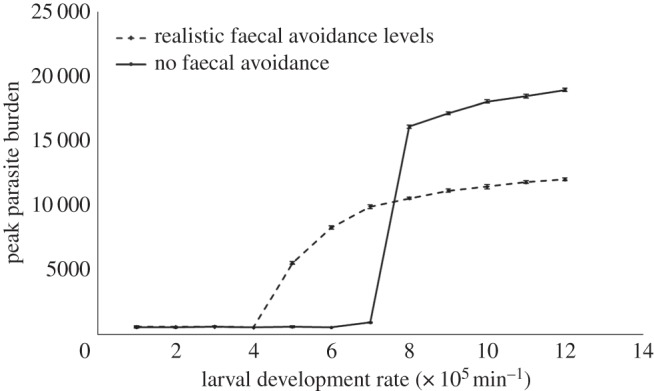
Peak parasite burdens over differing larval development rates for hosts with no faecal avoidance, and realistic levels of faecal avoidance behaviour (±s.d.).

The spatially explicit, population-level model was used to test the robustness of the tipping-point to spatial and individual-level effects. This second model also successfully reproduces the outbreak dynamics shown in figure 1 [29]. Nonlinear trends in the influence of climate-sensitive parameters (larval development and survival) on parasite burden were again evident in this spatial, individual-based model, showing that the presence of the tipping-point is robust to spatial- and individual-level effects. Although the qualitative patterns observed are independent of which model is used, it remains important to consider wider elements of the system. Figure 4 shows how grazing behaviour (in this example, host faecal avoidance) influences both the position of the tipping-point and magnitude of peak parasite burdens.

### 3.5. Changes in over-winter larvae survival

Climate change will also influence over-winter larval survival, and thus the concentration of infective larvae at the start of the grazing season. To explore the influence of over-winter survival on transmission dynamics, the spatial individual-based model was used, as the aggregation of larvae on pasture can have significant impacts on outbreak likelihood and magnitude [29]. Runs were initiated with low, medium and high numbers of infective larvae on the field, distributed over 0.3% of randomly selected patches, reflecting the typically aggregated distribution of free-living larvae [40]. The influence of initial contamination levels was explored over the full range of larval death and development rates, with hosts showing realistic faecal avoidance levels.

**Figure 5.**
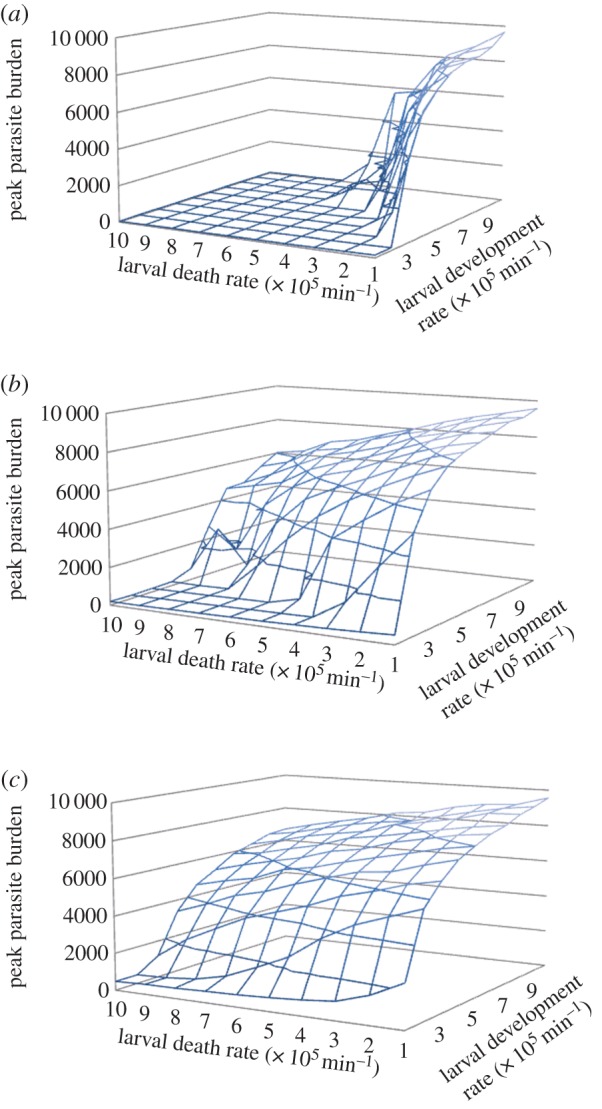
Influence of larvae development and death rates on parasite burden. Initial infective larvae concentration on pasture (*a*) low (12 000 per field), (*b*) medium (24 000 per field), and (*c*) high (48 000 per field).

The position of the tipping-point is influenced by the initial level of infective-larvae contamination on pasture at the start of the grazing year (figure 5), with higher initial levels giving rise to high-intensity outbreaks across a broader range of development and death rates.

## 4. Discussion

To explore the potential for climate change to influence outbreaks of livestock helminths, we determine the impact of variations in temperature-sensitive elements of the transmission process on host–parasite burdens.

### 4.1. Larval development

Temperature affects the development rates of parasites’ free-living stages, and simulations demonstrate that acceleration of larval development can lead to a nonlinear increase in parasite burdens, with a distinct tipping-point. This sudden rise is due to decreased development times allowing multiple generations of larvae to accumulate on pasture over one season, with the resultant feedback causing high-intensity outbreaks for parasites that would pose minimal risk under cooler climatic conditions. Consequently, a small change in temperature could result in a critical level being exceeded, leading to a sudden increase in parasite burdens with little warning. This could drive substantial increases in clinical cases of parasites that are currently widespread but at low intensities. The identification of this tipping-point provides a possible explanation for observed patterns of *H. contortus* infection in the UK (present at low levels across a wide thermal range, with occasional high-intensity outbreaks [6]), as inter-annual variability leads to the tipping-point being exceeded during warmer years. As summer temperatures continue to rise, high-intensity pathological *H. contortus* outbreaks are likely to occur in more years and across a greater geographical range. Once the tipping-point has been exceeded, outbreak intensity is increasingly dependent on the host immune response.

With the paucity of species-specific, long-term parasite intensity data across a range of temperatures, our predictions cannot be fitted for a specific host-pathogen system. However, the model has been shown to recreate empirically observed systems behaviour (i.e. seasonal parasite dynamics and host grazing behaviour), this allows us to explore broad expected patterns under novel conditions (i.e. changing parasite development rates). Rather than make species-specific predictions, this generalized model provides qualitative predictions of expected systems behaviour, and the dynamics are shown to be robust to the inclusion of wider transmission-scale processes. Further validation would be required to provide a quantitative result, but the potential for this mechanism to be operating in livestock systems has serious implications. There is a need for more data on changing patterns of parasite intensity under climate change, and this approach could inform targeting of data collection. Active collection of long-term surveillance data would not only allow model validation, but could also identify farms that are on the edge of the tipping-point as they begin to experience high parasite burdens during particularly warm years.

### 4.2. Larval death rate

Survival of the parasites free-living stages will also be influenced by changing climatic conditions. Increased minimum temperatures will reduce death rates of species of tropical origin, which are vulnerable to low temperatures (e.g. *H. contortus*). Conversely, increased temperatures are likely to increase death rates of temperate species which are impervious to prolonged cold conditions but vulnerable at high temperatures (e.g. *O. ostertagi*) [13]. Changes in larval death rates will impact on parasite transmission and cause shifts in the tipping-point's position (figure 3). The balance between development and death rates determines whether enough infective larvae are maintained for infections to perpetuate; climate change will sway the balance in opposing directions for different parasite species.

Owing to the predominant influence of temperature on larval development and survival, we have focused on this aspect of climate change. However, transmission is affected by wider abiotic elements, and climate change is about more than just rising temperatures. Further changes in rainfall patterns and moisture availability are also predicted, and relative humidity has been shown to influence larval survival and development [31,55,56]. Hence, changes in rates explored above could be seen as representative of changes in both temperature and rainfall patterns. However, many helminth species avoid the main impacts of reduced moisture availability as humidity inside host faeces is sufficient to allow hatching, and shelter the larvae from desiccation; so temperature remains the most important climatic determinant of larval levels [12,55].

### 4.3. The importance of livestock grazing behaviour and management

Using the spatially explicit, population model, it was demonstrated that the presence of the tipping-point is robust to spatial and individual-level effects. Although the indicative patterns observed are independent of model complexity, there is a need for models which incorporate wider elements of the system as the differences in quantitative effects give indications of how specific control and management strategies will influence outbreaks in a changing climate. For example, host grazing behaviour influences both the magnitude of peak parasite burdens and the position of the tipping-point (figure 4). Hence changes in the host's ability to demonstrate natural grazing behaviours, which is affected by its physiological state and management decisions [23,50], will influence parasite outbreaks. The importance of faecal avoidance on outbreak trends is due to changes in the timing of ingestion of free-living parasites. Grazing species have high faecal avoidance behaviours and are more at risk from pathogens that develop slowly in the environment and reach peak infectivity when faeces have decayed and grass has grown tall. By contrast, in the absence of faecal avoidance behaviour, hosts are at increased risk from parasites which develop quickly on pasture, while effectively diminishing risk from slow developing parasites by ingesting them before they become infective.

### 4.4. Changes in over-winter larvae survival

The contamination levels of infective larvae on pasture at the start of the grazing season influence the position of the tipping-point (figure 5). An increase in pasture contamination of larvae leads to the tipping-point being reached for a broader range of death and development rates. Climate change will have contrasting effects on the over-wintering potential of different parasite species. For temperate species that can survive cold winters, warmer temperatures could decrease over-winter survival [1]. This is owing to temperatures accelerating the metabolic rate of infective larvae, depleting their finite energy reserves as protective sheaths prevent feeding. However, decreased survival on pasture could be counteracted by lengthening of the grazing season; parasites may not have to survive as long in a host free environment. For tropical species, the decrease in frosts, combined with an extended grazing season, could enable survival of parasites on pasture over winter. There is also potential for feedback between climate-driven changes in outbreaks within grazing seasons and the size of over-wintering larval populations, owing to larger populations at the start of winter. However, the concentration of infective larvae on pasture grazed by naive hosts can be managed through rotational grazing or larvicide application, allowing the influence of temperature on development to be counteracted through livestock management and control strategies. If rising temperatures push parasite development rates far beyond critical thresholds, and complete alleviation of outbreaks is unfeasible, increasing the ability of hosts to acquire resistance (e.g. though genetic selection or improved nutrition) could dampen outbreak intensity, as above the tipping-point peak parasite burden is governed by host immunity.

## 5. Conclusion

Our results indicate that climate change can lead to nonlinear responses in infection dynamics, such that minor alterations in temperature around critical thresholds could cause dramatic shifts in outbreak intensity. This could lead to an increase in the frequency and geographical range of pathological cases for pathogens that are currently widespread but at low incidence levels. The relationship between survival and development of the parasites’ free-living stages, over-winter larval survival and behavioural characteristics of the host are pivotal determinants of outbreak intensity.

## Data Accessibility

There are no archived data to accompany this paper as the work is based on a mechanistic, process-based model, using data extracted from previously published papers for model parametrization.
